# What are the optimal pharmacokinetic/pharmacodynamic targets for β-lactamase inhibitors? A systematic review

**DOI:** 10.1093/jac/dkae058

**Published:** 2024-03-09

**Authors:** Getnet M Assefa, Jason A Roberts, Solomon A Mohammed, Fekade B Sime

**Affiliations:** Centre for Clinical Research, Faculty of Medicine, The University of Queensland, Brisbane, QLD, Australia; Department of Pharmacy, College of Medicine and Health Sciences, Wollo University, Dessie, Ethiopia; Centre for Clinical Research, Faculty of Medicine, The University of Queensland, Brisbane, QLD, Australia; Pharmacy Department, Royal Brisbane and Women’s Hospital, Brisbane, QLD, Australia; Department of Intensive Care Medicine, Royal Brisbane and Women’s Hospital, Brisbane, QLD, Australia; Herston Infectious Disease Institute (HeIDI), Metro North Health, Brisbane, QLD, Australia; Division of Anaesthesiology Critical Care Emerging and Pain Medicine, Nimes University Hospital, University of Montpellier, Nimes, France; Centre for Clinical Research, Faculty of Medicine, The University of Queensland, Brisbane, QLD, Australia; Department of Pharmacy, College of Medicine and Health Sciences, Wollo University, Dessie, Ethiopia; Centre for Clinical Research, Faculty of Medicine, The University of Queensland, Brisbane, QLD, Australia

## Abstract

**Background:**

Pharmacokinetic/pharmacodynamic (PK/PD) indices are widely used for the selection of optimum antibiotic doses. For β-lactam antibiotics, *fT*_>MIC_, best relates antibiotic exposure to efficacy and is widely used to guide the dosing of β-lactam/β-lactamase inhibitor (BLI) combinations, often without considering any PK/PD exposure requirements for BLIs.

**Objectives:**

This systematic review aimed to describe the PK/PD exposure requirements of BLIs for optimal microbiological efficacy when used in combination with β-lactam antibiotics.

**Methods:**

Literature was searched online through PubMed, Embase, Web of Science, Scopus and Cochrane Library databases up to 5 June 2023. Studies that report the PK/PD index and threshold concentration of BLIs approved for clinical use were included. Narrative data synthesis was carried out to assimilate the available evidence.

**Results:**

Twenty-three studies were included. The PK/PD index that described the efficacy of BLIs was *fT*_>CT_ for tazobactam, avibactam and clavulanic acid and *f*AUC_0–24_/MIC for relebactam and vaborbactam. The optimal magnitude of the PK/PD index is variable for each BLI based on the companion β-lactam antibiotics, type of bacteria and β-lactamase enzyme gene transcription levels.

**Conclusions:**

The PK/PD index that describes the efficacy of BLIs and the exposure measure required for their efficacy is variable among inhibitors; as a result, it is difficult to make clear inference on what the optimum index is. Further PK/PD profiling of BLI, using preclinical infection models that simulate the anticipated mode(s) of clinical use, is warranted to streamline the exposure targets for use in the optimization of dosing regimens.

## Introduction

β-Lactam (BL) antibiotics are one of the widely used classes of antimicrobials to battle the war against bacterial infections. Even though these antibiotics have been very effective in saving millions of lives, soon after their introduction into clinical practice, their effectiveness progressively reduced due to the development of resistance, mainly mediated by β-lactamase enzymes produced by Gram-negative bacteria.^1,2^ β-Lactamase enzymes are usually classified based on the sequence of their amino acids into Classes A, B, C and D. Those that bind to substrates through a serine active site (Class A, C and D) are commonly known as serine β-lactamases and those that utilize a metal (zinc) ion to bind with the BL ring of their substrates (Class B) are called MBLs.^3–5^

A widely used strategy to circumvent resistance due to β-lactamase enzyme expression is combining BL antibiotics with β-lactamase inhibitors (BLIs), which bind to the enzymes to thwart the hydrolysis of BL antibiotics by the enzymes.^6,7^ BLIs are classified into first-generation BLIs (clavulanic acid, sulbactam and tazobactam), which inactivate Class A and some Class C serine β-lactamases, and second-generation BLIs (avibactam, relebactam and vaborbactam), which inhibit Class A, Class C and some Class D serine β-lactamases.^7,8^ The use of these inhibitors with BL antibiotics allows the restoration of the therapeutic efficacy against MDR bacteria and their use has become a trend in drug discovery. In addition to combinations of amoxicillin/clavulanic acid, ampicillin/sulbactam and piperacillin/tazobactam, which have been in use for decades, recently ceftazidime/avibactam, meropenem/vaborbactam, imipenem/cilastatin/relebactam and ceftolozane/tazobactam have been approved for clinical use.^9–12^ A number of other combinations of novel BLIs with existing BL antibiotics are currently undergoing development.^13–17^ The effectiveness of the combination depends on the inherent stability of the stand-alone BL against β-lactamase enzymes, the potency of the BLI and the adequacy of the BL/BLI amounts contained in the combination.^18,19^

The selection of optimal antibiotic dose is guided by dose–exposure–response analysis, in which exposure is often described by pharmacokinetic/pharmacodynamic (PK/PD) parameters.^20–25^ PK/PD parameters are usually determined by either *in vivo* or *in vitro* preclinical infection model studies.^26^ These models use dose-ranging and dose-fractionation experiments to determine the PK/PD index that correlates exposure to the efficacy of the antibiotic. *In silico* methods may also be applied to predict PK/PD exposure metrics using advanced mathematical models built from *in vitro* or animal model experimental data.^27,28^ The three most common PK/PD exposure metrics include: the ratio of the area under the free drug concentration–time curve at 24 h to MIC (*f*AUC_0–24_/MIC); the ratio of the maximum free drug concentration to MIC (*fC*_max_/MIC); and the fraction of dosing interval that the free drug concentration remains above MIC (*fT*_>MIC_).^29,30^

For BL antibiotics, *fT*_>MIC_ best relates antibiotic exposure to efficacy, whereby a magnitude of 40%–70% *fT*_>MIC_ is required to produce a bactericidal effect.^31,32^ This time-dependent PK/PD parameter for BLs is traditionally determined in a fixed ratio combination with BLIs, optimized using simpler static experiments, and is used to guide the dosing of BL/BLI combinations, often without considering any PK/PD exposure requirements for the BLIs. Most BLIs lack sufficient intrinsic antibacterial activity and delineating the PK/PD index that describes the efficacy of these agents is relatively complex. The PK/PD index of BL/BLI combinations should ideally consider the contribution of the inhibitor in the combination and be able to specify the magnitude of the exposure measure necessary for the efficacy of the BLI. However, identifying a single PK/PD index that describes the efficacy of both the BL antibiotic and the BLI is challenging.^33^ One strategy to tackle this problem might be optimizing the exposure of BLIs using a separate PK/PD exposure metric required to protect the combined BL. However, data on the optimal PK/PD targets of BLIs are limited. Therefore, this systematic review aims to assess existing literature and assimilate data on the PK/PD targets of clinically approved BLIs.

## Methods

### Study protocol

This systematic review was conducted following the Preferred Reporting Items for Systematic Review and Meta-analysis (PRISMA) guidelines.^34^

The protocol for this review was registered at the international prospective register of systematic reviews (PROSPERO) with registration number CRD42023440787 (https://www.crd.york.ac.uk/prospero/display_record.php? ID=CRD42023440787).

### Data sources and search strategy

Literature was searched online through PubMed, Embase, Web of Science, Scopus and Cochrane Library databases up to 5 June 2023. The search strategy was designed based on two concepts: (i) BLI: clavulanic acid, sulbactam, tazobactam, avibactam, relebactam and vaborbactam; and (ii) target exposure: target concentration, threshold concentration, PK/PD index, PK/PD target, PK/PD ratio, pharmacodynamics, IC_50_, effective concentration, critical concentration and PD index. Appropriate indexing terms, truncations and Boolean operators were used to make the search exhaustive. In addition, relevant articles were searched from reference lists of retrieved articles. The search was limited to the English language. The detailed search terms used for each database are presented in Table S1 (available as Supplementary data at *JAC* Online).

### Inclusion and exclusion criteria

Inclusion criteria were: for BLIs, those approved for clinical use before 5 June 2023; for study design, preclinical (*in vitro*, *in vivo*, *in silico*) and clinical studies that reported the PK/PD index and/or concentration of BLI that protected the companion BL antibiotic; for outcome, the exposure measure (PK/PD index) associated with the efficacy of BLI and the magnitudes of the exposure measures (PK/PD target) necessary for the efficacy of BLI; and for language, articles published in the English language.

Exclusion criteria were: studies that had not determined the threshold concentration or PK/PD exposure target of BLIs; and reviews, guidelines, protocols, brief communications, books, letters to the editor, errata and conference abstracts.

### Study selection

The citations retrieved from various databases were imported to Covidence software (www.covidence.org). The software identified, recorded and removed duplicates, and careful visual assessment was undertaken to remove duplicates that were not detected by the software. Two authors (G.M.A. and S.A.M.) independently assessed the titles, abstracts and full documents of each record using the predefined selection criteria. Disagreements between the two authors during the screening process were solved through discussion.

### Quality assessment tool

The quality assessment tool was customized from previous studies.^35–37^ The tool consisted of 15 items to assess the quality of *in vitro* and *in vivo* studies (Table S2) and 13 items to assess the quality of *in silico* studies (Table S3). The quality of the included papers was assessed by G.M.A. and S.A.M. and discrepancies were solved by discussion.

### Data extraction

The data extraction form was prepared in Microsoft Excel and data were extracted from the included studies’ texts, tables and graphs. The data extracted included name of the author, year of publication, methods used, BLI studied, companion BL, species and strain of the bacteria, β-lactamase enzyme expressed, initial concentration of the bacteria, duration of the experiment, PK/PD index that described the BLI efficacy and the magnitudes of the exposure measures necessary for net bacterial stasis, 1 log and 2 log kills.

### Outcome measurement

The primary outcome of this systematic review was the PK/PD index associated with the efficacy of the BLI and the magnitude of this exposure necessary for the efficacy of the BLI.

## Results

### Search findings

**Figure 1.**
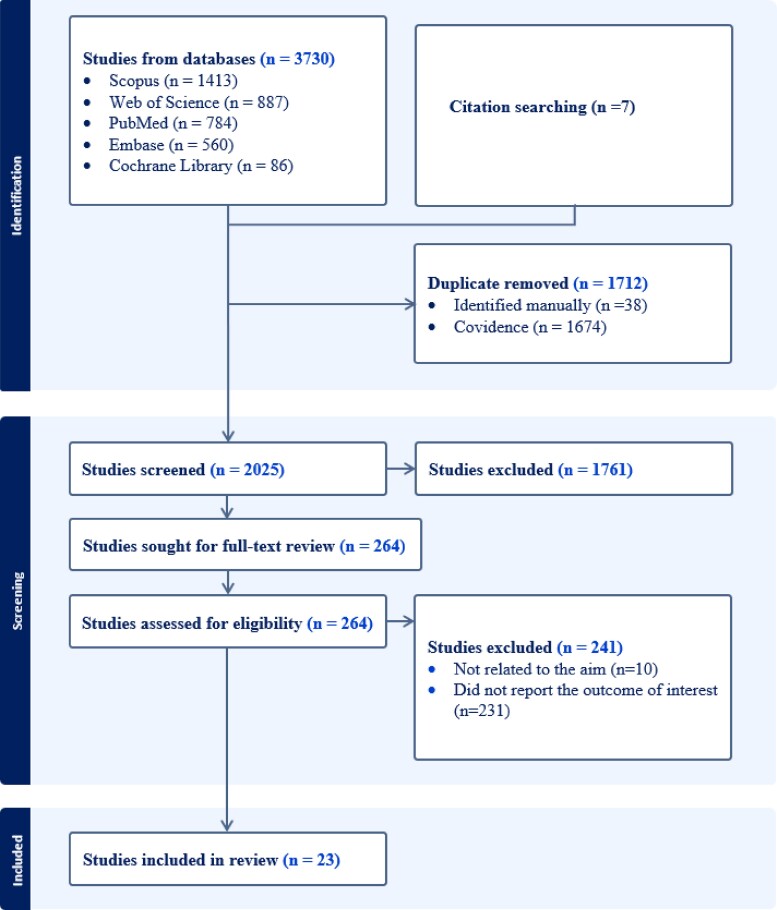
PRISMA flow diagram showing the selection process of identified studies. This figure appears in colour in the online version of *JAC* and in black and white in the print version of *JAC*.

A total of 3737 studies were retrieved from databases and citation searching, from which 1712 duplicates were removed. Of 2025 studies that underwent title and abstract screening, 264 passed for full document review. Finally, 23 studies were included in the systematic review. The whole study selection process is presented in the PRISMA flow diagram (Figure 1).

### Quality assessment results

From 23 preclinical studies, 10 studies reported all quality assessment items. The least reported items of the quality assessment tool for the *in vitro* and *in vivo* studies were appropriate methodology for dose-fractionation/ranging studies, and magnitude of exposure measure associated with the efficacy of BLI, which were not reported by 6 and 7 studies, respectively, as shown in Table S4. Additionally, in the case of *in silico* studies, two out of the three studies did not carry out prospective validation of their *in silico* predictions, as shown in Table S5.

### Study characteristics

A total of 23 studies were included in this systematic review.^38–60^ From these, 11 studies used *in vitro* infection models,^39,43,44,46–48,52,56–59^ 7 studies used *in vivo* infection models,^38,40,49–51,53,60^ and 2 studies used both *in vitro* and *in vivo* infection models^45,54^ to evaluate the optimal concentration of BLI necessary to protect the companion BL antibiotic. Three of the studies were based on *in silico* methods, using mathematical modelling to determine the outcome of interest.^41,42,55^ Tazobactam and avibactam were the most widely studied BLIs, as shown in Table 1.

**Table 1.** Study characteristics

BLI studied	Companion BL	Infection model	Study duration	Bacterial species	β-Lactamase enzyme expressed	Baseline bacterial concentration	References
Clavulanic acid	Ceftibuten	Murine thigh	24 h	*E. coli* & *K. pneumoniae*	CTX-M gp1, TEM-WT, CTX-M gp9, SHV-ESBL, SHV-WT,	10^6^ cfu/thigh	^38^
		*In vitro* chemostat	24 h	*K. pneumoniae* & *E. coli*	CTX-M-55, CTX-M-15, CTX-M-14, TEM, SHV-12	10^6^ cfu/mL	^46^
Sulbactam		Murine thigh and lung	24 h	*A. baumannii*	—	10^6^ cfu/mL	^60^
Tazobactam	Piperacillin	*In vitro* model	24 h	*E. coli*	CTX-M-15 (low-, moderate- & high-level expression)	1.0 × 10^6^ cfu/mL	^52^
		Murine thigh	24 h	*E. coli*	TEM-1 expressed differently	10^4^ cfu/thigh	^53^
		HFIM	72 h	*K. pneumoniae* & *E. coli*	CTX-M-15 & SHV-12	10^6^ cfu/mL	^39^
	Ceftolozane	Mouse thigh	24 h	*E. coli* & *K. pneumoniae*	CTX-M15, SHV-1, OXA-1, TEM-1, SHV-5, TEM-84, SHV-11, CTX-M1, CTX-M2, CTX-M14	1 × 10^6^ cfu/thigh	^50^
		*In vitro* model	24 h	*E. coli* & *K. pneumoniae*	AmpC, CTX-M-15	1 × 10^6^ cfu/mL	^56^
		*In vitro* model	24 h	*E. coli*	CTX-M-15 (low, moderate & high level)	1 × 10^6^ cfu/mL	^57^
	Cefepime	Murine thigh	24 h	*E. coli*, *K. pneumoniae* & *E. cloacae*	CTX-M-9, OXA-1, CTX-M-15, TEM-1, CTX-M-14, TEM-84, SHV-11, CTX-M-1, CTX-M-39, AmpC	2–3 × 10^6^ cfu/thigh	^51^
		*In vitro* model	24 h	*E. coli* & *K. pneumoniae*	CTX-M-15, TEM-1, OXA-1/30, SHV-28, OXA-2, SHV-1	1 × 10^6^ cfu/mL	^58^
Avibactam	Ceftazidime	Murine thigh and lung	24 h	*P. aeruginosa*	AmpC, TEM-24	5 × 10^5^–10^8^ cfu/thigh	^40^
		HFIM	24–48 h	*K. pneumoniae*, *E. cloacae* & *C. freundii*	AmpC, CTX-M-15, TEM-1, OXA-1, SHV-11, SHV-5, TEM-10, KPC-3,	1–3 × 10^5^ cfu/mL	^43^
		HFIM	10 days	*K. pneumoniae*	*bla*_KPC-2_, *bla*_SHV-11_, *bla*_SHV-12_ and *bla*_OXA-9_	>10^8^ cfu/mL	^44^
		*In silico* model	—	*P. aeruginosa*	—	—	^55^
	Ceftaroline, ceftazidime	*In vitro* model	24 h	*E. coli*, *E. cloacae* & *K. pneumoniae*	CTX-M AmpC and KPC	—	^48^
	Ceftaroline	HFIM	10 days	*K. pneumoniae*	KPC-2, SHV-27, and TEM-1	1.5 × 10^7^–1.5 × 10^8^ cfu/mL	^47^
	Aztreonam	HFIM	24 h	*E. coli* & *K. pneumoniae*	NDM-1, CTX-M-15, OXA-1, SHV-1, TEM-1, SHV-11, CMY-6, SHV-2a, TEM-208, OXA-2, CMY-4, OXA-9, CMY-42, CMY-6	10^6^ cfu/mL	^54^
		Murine thigh	24 h	*E. coli* & *K. pneumoniae*	NDM-1, CTX-M-15, OXA-1, TEM-1, SHV-11, SHV-2a, CMY-6	10^6^ cfu/thigh	
Relebactam	Imipenem	*In silico*	—	*K. pneumoniae*	KPC-2	10^5^ cfu/mL	^41^
		*In silico*	—	*P. aeruginosa*	—	—	^42^
		Murine thigh	24 h	*P. aeruginosa & K. pneumoniae*	AmpC & KPC	5 × 10^6^ cfu/thigh	^49^
		HFIM	72 h	*P. aeruginosa*, *K. pneumoniae*, *E. coli*, *Klebsiella oxytoca*, *Serratia marcescens*	KPC-2, TEM, SHV, KPC-3, CTX-M-14, DHA, CTX-M-3, CTX-M-15, CMY-2, TEM-OSBL, CTX-M-14, CMY-140, KPC-6, KPC-11	10^6^ cfu/mL	^59^
Vaborbactam	Meropenem	HFIM	32 h	*K. pneumoniae*, *E. coli* & *E. cloacae*	KPC-2, KPC-3, SHV-11, TEM-1, CTX-M-15, SHV-12, CTX-M-14, LEN-17, SHV-12, TEM, SHV, OXA-10	∼10^8^ cfu/mL	^45^
		Mouse thigh	24 h	*K. pneumoniae* & *E. cloacae*	KPC-2, 3, SHV-11, TEM, TEM-1, LEN-17, SHV, SHV-12	1 × 10^6^ cfu/mL	

Most studies used *Klebsiella pneumoniae*, *Escherichia coli* and *Pseudomonas aeruginosa*, each expressing different β-lactamase enzymes. The initial inoculum concentration was approximately 10^6^ cfu/mL (*in vitro* studies) or 10^6^ cfu/thigh (*in vivo* studies) (Table 1).

In studies that explored different PK/PD indices for BLI, the fraction of the dosing interval that the free drug concentration is above a threshold concentration (*fT*_>CT_) was identified as the best PK/PD index for tazobactam,^50–53,57^ avibactam^40,44,54,55^ and clavulanic acid.^38,46^ Consistent with this, Louie *et al*^47^ demonstrated that *fT*_>CT_ adequately describes the exposure–response relationship for avibactam. The PD portion of the PK/PD index of these BLIs, due to lack of intrinsic antibacterial activity, is usually represented by a C_T_ for inhibition instead of MIC.^33^ The C_T_ is the lowest concentration of BLI that must be maintained to achieve sufficient β-lactamase inhibition to protect the hydrolysis of the companion BL.^61^ The PK/PD index that described the efficacy of relebactam^42,49,59^ and vaborbactam^45^ was *f*AUC_0–24_/MIC at the MIC of imipenem and meropenem potentiated with each BLI, respectively, as depicted in Table 2.

**Table 2.** The PK/PD index associated with efficacy and the magnitude of exposure for the efficacy of the BLIs

BLI studied	Companion BL	Infection models used	PK/PD index	Threshold concentration	The magnitude (%) of the exposure required for			References
					Stasis	1 log kill	2 log kill	
Clavulanic acid	Ceftibuten	Murine thigh	*fT*_>CT_	0.5 mg/L	21	92	—	^38^
		*In vitro* chemostat	*fT*_>CT_	0.06–1 mg/L	31–52	48–92	—	^46^
Sulbactam		Murine thigh	*fT*_>MIC_	—	21	33	44	^60^
		Murine lung	*fT*_>MIC_	—	20	25	29	
Tazobactam	Piperacillin	*In vitro*	*fT*_>CT_	0.25, 0.5 and 2 mg/L for the low-, moderate- and high-level CTX-M-15-producing strains	45	63	85	^52^
		Murine thigh	*fT*_>CT_	0.5 mg/L and 2 mg/L for the two strains of *E. coli* with different TEM-1	42	56	—	^53^
		HFIM	*fT*_>MICi_^a^	—	55–60	—	—	^39^
	Ceftolozane	Mouse thigh	*fT*_>CT_	0.5 mg/L	28.2 (17.5–45.8)	44.4% (26.6–54.7)	—	^50^
		*In vitro*	*fT*_>CT_	0.5 × MIC	66	77	90	^56^
		*In vitro*	*fT*_>CT_	0.05 mg/L (low & moderate), 0.25 mg/L (high BLE)	35	50	70	^57^
	Cefepime	Murine thigh	*fT*_>CT_	0.25 mg/L	24.6 (11.4–36.3)	39.7 (16.5–54.0)	—	^51^
		*In vitro*	*fT*_>CT_	0.125 × MIC	22	53	—	^58^
Avibactam (NXL104)	Ceftazidime	Murine thigh	*fT*_>CT_	1 mg/L	31 (14.1–62.5)	46.9 (32.9–67.2)	—	^40^
		Murine Lung	*fT*_>CT_	1 mg/L	13.6 (0–21.4)	14.3 (0–22.4)	—	
		HFIM		0.3 mg/L	—	—	—	^43^
		HFIM	*fT*_>CT_	4 mg/L	—	—	—	^44^
		*In silico*	*fT*_>CT_	1 mg/L	20–60	≥50	—	^55^
		*In vitro*	*f*AUC_0–24_	—	—	—	—	^48^
	Ceftaroline	HFIM	*fT*_>CT_	4 mg/L	62–80	—	—	^47^
		*In vitro*	*f*AUC_0–24_	—	—	—	—	^48^
	Aztreonam	HFIM	*fT*_>CT_	2–2.5 mg/L	34–56.4	37.7–58.2	—	^54^
		Murine thigh		2–2.5 mg/L	23–25	35–40		
Relebactam	Imipenem	*In silico*	*fT*_>MICi_^a^	—	>69	—	—	^41^
		*In silico*	*f*AUC_0–24_/MIC	—	2.7	4.7	7.5	^42^
		Murine thigh	*f*AUC_0–24_/MIC	—	5.2	—	—	^49^
		HFIM	*f*AUC_0–24_/MIC	—	8.2	12	18	^59^
Vaborbactam	Meropenem	HFIM	*f*AUC_0–24_/MIC	—	12	18	25	^45^
		Mouse thigh	*f*AUC_0–24_/MIC	—	9	38	220	

BLE, β-lactamase expression.

^a^Indicates the PK/PD of the BL/BLI combination.

### Study outcomes

#### Clavulanic acid

The combination of clavulanic acid with ceftibuten is in development for the treatment of complicated urinary tract infection (cUTI) caused by ESBL-producing Enterobacterales. The PD of this combination was evaluated using *in vitro* one-compartment^46^ and *in vivo* murine thigh infection models,^38^ against different strains of *K. pneumoniae* and *E. coli* that express different β-lactamase enzymes. The PK/PD index that best described the efficacy of clavulanate when combined with ceftibuten was *fT*_>CT_ in both models. The C_T_ required for the efficacy of clavulanate when combined with ceftibuten was 0.5 mg/L in a murine thigh infection model,^38^ and ranged from 0.006 to 1 mg/L in an *in vitro* infection model, depending on the bacterial strain.^46^ The magnitude of *fT*_>CT_ required to achieve net bacterial stasis and 1 log_10_ kill were 21% and 92%, respectively, in an *in vivo* study,^38^ but varied according to the test strain in an *in vitro* study (31% and 48% for *E. coli*; and 52% and 92% for *K. pneumoniae* for net stasis and 1 log_10_ kill, respectively).^46^

#### Sulbactam

Unlike other BLIs, sulbactam has inherent antibacterial activity against *Acinetobacter baumannii* strains. Even though the PK/PD index that described the efficacy of sulbactam with a companion BL is not clearly reported, the PK/PD of sulbactam alone against *A. baumannii* was evaluated by Yokoyama *et al.* using *in vivo* murine thigh and lung infection models.^60^ In both models, the relationship between the efficacy of sulbactam against *A. baumannii* ATCC 19606 and the common PK/PD indices (*fT*_>MIC_, *f*AUC_0–24_/MIC and *fC*_max_/MIC) was evaluated. The antibacterial activity of sulbactam was best correlated with *fT*_>MIC_ with R^2^ values of 0. 95 and 0.96 in the thigh and lung infection models, respectively. The *fT*_>MIC_ required for net stasis, 1 and 2 log_10_ cfu reductions were 21%, 33% and 44% in the thigh infection model and 20%, 25% and 29% in the lung infection model, respectively. They also reported sulbactam was sufficiently bactericidal when an *fT*_>MIC_ of >60% and >40% is achieved in the thigh and lung infection models, respectively. However, the PK/PD index associated with sulbactam’s efficacy when combined with BLs is not clearly stated. For instance, studies done by Alexov *et al*.,^62^ and Lister *et al.*^63^ reported that the efficacy of the combination is maintained when the concentration of the sulbactam is above the enzyme inhibitory concentration. According to these studies, the enzyme inhibition efficacy of sulbactam can be maximized by prolonging *fT*_>CT_; however, they didn’t report the specific C_T_ required for its action.

#### Tazobactam

Tazobactam is one of the most studied BLIs. Of the included studies, eight of them studied the PK/PD of tazobactam in combination with piperacillin, ceftolozane and cefepime by using different *in vitro* and *in vivo* infection models.^39,50–53,56–58^

##### Piperacillin/tazobactam

The PK/PD of tazobactam, combined with piperacillin, was evaluated by one *in vivo* and two *in vitro* studies. Nicasio *et al.*^52^ used an *in vitro* infection model and isogenic CTX-M-15-producing *E. coli* that transcribed different levels of *bla*_CTX-M-15_ to describe the PK/PD index associated with tazobactam efficacy when combined with piperacillin. Data from dose-ranging and dose-fractionation studies showed that *fT*_>CT_ is the PK/PD index that best correlates with the enzyme inhibitory efficacy of tazobactam when combined with piperacillin, irrespective of the transcription level of the enzyme (r^2 ^= 0.839), but the C_T_ varies from 0.25 to 2 mg/L based on the transcription level of the enzyme, as shown in Table 2. The magnitude of *fT*_>CT_ (at C_T_ from 0.25 to 2 mg/L) required for tazobactam for bacterial stasis and a 1 and 2 log_10_ bacterial kill were 45%, 63% and 85%, respectively.^52^

A study by Rodriguez *et al.* used a neutropenic murine thigh infection model to assess the efficacy of the piperacillin/tazobactam combination on two isogenic strains of *E. coli* that express TEM-1 differentially. *fT*_>CT_ is the PK/PD index that best described the efficacy of tazobactam with C_T_ values of 0.5 and 2 mg/L for the two strains of *E. coli* that express TEM-1 differently. The mean *fT*_>CT_ required for net bacterial stasis and 1 log_10_ kill was 42% and 56%, respectively.^53^

Another study identified piperacillin MIC reduction with an increased concentration of tazobactam. This study showed that the clinical regimen of piperacillin/tazobactam (4 g/0.5 g q8h) was unable to suppress the bacterial load of the four tested strains using a hollow-fibre infection model (HFIM). However, increasing the dose of tazobactam to 1 and 1.5 g suppressed the growth of SHV-12-producing *E. coli* and CTX-M-15-producing *K. pneumoniae* strains, respectively. The parameter *fT*_>MICi_, where MICi represents the impact of varying concentrations of the BLI on the MIC of the companion BL antibiotic, serves as a modified PK/PD index for BL/BLI combinations. This index measures the exposure of the BL, piperacillin, at variable concentrations of the inhibitor, tazobactam. For the CTX-M-15-producing *K. pneumoniae* strain (using 1.5 g of tazobactam), a 55% *fT*_>MICi_ of piperacillin (4 g) was associated with stasis, while for the SHV-12-producing *E. coli* strain (with 1 g of tazobactam), the corresponding value was 60%. However, increasing the tazobactam dose up to 4 g did not result in effective bacterial suppression for the other two CTX-M-15-producing *K. pneumoniae* strains.^39^

##### Ceftolozane/tazobactam

The study by VanScoy *et al.*^57^ used a one-compartment *in vitro* infection model to evaluate the PK/PD of ceftolozane/tazobactam against an isogenic *E. coli* strain that produces *bla*_CTX-M-15_. This study evaluated the effect of administering tazobactam at different dosing intervals (6, 8, 12 and 24 h) with similar total daily doses and the more frequent administrations of tazobactam (q6h and q8h) were shown to enable reduction of the bacterial load by more than 2 log_10_ cfu/mL at 24 h regardless of the β-lactamase enzyme construct.^57^ The relationship between the different PK/PD parameters and change in log_10_ cfu at 24 h was evaluated and *fT*_>CT_ was identified as the exposure measure that best predicted the efficacy of tazobactam (r^2 ^= 0.938) with a C_T_ of 0.05 mg/L for the low and moderate (r^2 ^= 0.975 and 0.972, respectively) and 0.25 mg/L (r^2 ^= 0.914) for the high β-lactamase gene transcription levels. The *fT*_>CT_ of 0.05 mg/L (for low and moderate β-lactamase expression) and 0.25 mg/L (for high β-lactamase expression) necessary for net bacterial stasis, 1 and 2 log cfu reduction at 24 h was 35%, 50% and 70% of the dosing interval, respectively.^57^

As shown by VanScoy *et al.*,^57^ in the study described above, the C_T_ of tazobactam was variable for a single isolate that expressed β-lactamase enzyme at different levels. To further understand this concept, the same group of researchers evaluated the relationship between tazobactam enzyme inhibition efficacy and *fT*_>CT_ using four different *E. coli* isolates.^56^ This study observed that the relationship between *fT*_>CT_ and change in log_10_ cfu from baseline at 24 h was well described by a sigmoid function (r^2^ = 0.90–0.99) and the estimated C_T_ for each isolate ranged from 0.5 to 4 mg/L. Moreover, they pooled the C_T_ of each *E. coli* isolate together and showed that the efficacy of tazobactam is expressed by the MIC of ceftolozane/tazobactam for each isolate multiplied by 0.5 (*fT*_>0.5_ _×_ _MIC_), which also performs well for the extended datasets that include four *E. coli* and three *K. pneumoniae* isolates. The tazobactam *fT*_>CT_ at 0.5 × MIC of ceftolozane/tazobactam required for net bacterial stasis, 1 log cfu/mL kill and 2 log_10_ cfu/mL kill at 24 h were 66%, 77% and 90% of the dosing interval.^56^

The PD of tazobactam in combination with ceftolozane was further evaluated by an *in vivo* study using a neutropenic mouse thigh infection model.^50^ This study also identified *fT*_>CT_ as the PK/PD index that best predicted the efficacy of tazobactam when combined with ceftolozane, and the C_T_ required for efficacy ranged from 0.5 to 2 mg/L, depending on the strain. The mean *fT*_>CT_ at a C_T_ of 0.5 mg/L tazobactam responsible for net bacterial stasis and a 1 log kill were 28.2% and 44.4%, respectively, for all tested strains.^50^

##### Cefepime/tazobactam

Two studies evaluated the PK/PD of cefepime/tazobactam combinations.^51,58^ The study conducted by Melchers *et al.*^51^ used a neutropenic murine thigh infection model to evaluate the efficacy of a cefepime/tazobactam combination against ESBL-producing Enterobacterales. Different dosage regimens of tazobactam (12–768 mg/kg/day every 2, 3, 4, 6 and 12 h) was fractionated against six ESBL-producing isolates, and *fT*_>CT_ was the PK/PD index that best correlated with the efficacy of tazobactam. To determine the C_T_, a fixed daily dose of tazobactam (seven thresholds from 0.0625 to 4 mg/L) was co-administered with 1 mg/kg cefepime. The mean C_T_ corresponding to the highest R^2^ for the six strains was 0.25 mg/L. The magnitude of *fT*_>CT_ at a C_T_ of 0.25 mg/L required for net bacterial stasis and 1 log_10_ kill ranged from 11.4% to 36.3% and 16.5% to 54.0%, respectively.^51^

In the other study done by VanScoy *et al*.,^58^ they developed a translational relationship with the MIC characterizing the PK/PD index of tazobactam when combined with cefepime against ESBL-producing Enterobacterales of different MICs. They proposed *fT*_>0.125_ _×_ _MIC_ as the PK/PD index of tazobactam when combined with cefepime, with the MIC of cefepime/tazobactam measured by conventional methods. The magnitudes of *fT*_>0.125_ _×_ _MIC_ necessary for bacterial suppression and a 1 log_10_ kill at 24 h were 22% and 53%, respectively.

#### Avibactam

The PK/PD of avibactam (formerly known as NXL104 and AVE1330A) is the most studied among the second-generation BLIs. The clinical use of avibactam in combination with ceftazidime is FDA-approved, while its combination with aztreonam is currently under development.^9,13^

##### Ceftazidime/avibactam

The PK/PD of avibactam in combination with ceftazidime was studied using *in vivo*,^40^  *in vitro*^43,44,48^ and *in silico* methods.^55^

The *in vivo* method used murine thigh and lung infection models inoculated with different strains of *P. aeruginosa* to study the PK/PD index that described the efficacy of avibactam when combined with ceftazidime. After the dose-fractionation study, both the thigh and lung infection models identified *fT*_>CT_ as the PK/PD index that best described the efficacy of avibactam when combined with ceftazidime, with a C_T_ of 1 mg/L. The magnitude of *fT*_>CT_ at a C_T_ of 1 mg/L required to achieve net bacterial stasis and 1 log_10_ kill is higher in the thigh infection model (31% and 46.9%, respectively) than the lung infection model (13.6% and 14.3%, respectively), even though the two models were inoculated with a similar number of bacteria.^40^

Coleman *et al*.^43^ and Drusano *et al.*^44^ used an HFIM to study the PD of ceftazidime and avibactam. The study by Coleman *et al.* used different bacterial species of Enterobacterales (*K. pneumoniae*, *Enterobacter cloacae* and *Citrobacter freundii*) at a low inoculum concentration (10^5^ cfu/mL) and Drusano *et al.* used two strains of *K. pneumoniae* at higher inoculum concentrations (>10^8^ cfu/mL). Drusano *et al.* identified *fT*_>CT_ as the PK/PD index that described the efficacy of avibactam when combined with ceftazidime. The C_T_ required for the efficacy of avibactam was identified to be 4 mg/L by Drusano *et al*.;^44^ however, it was not identified by Coleman *et al.* (suggested to be less than 0.3 mg/L).^43^ The *fT*_>CT_ required to achieve net bacterial stasis, 1 or 2 log kill was not determined by either study.

An *in vitro* infection model was used by MacGown *et al.* to study the PD of avibactam in combination with ceftazidime and ceftaroline. The study applied a series of dose-fractionation experiments to identify the PK/PD index that described avibactam efficacy when combined with ceftazidime and ceftaroline against CTX-M-producing *E. coli*, AmpC-hyperproducing *E. cloacae* and KPC-producing *K. pneumoniae*. Unlike other studies discussed above, AUC_0–24_ and *C*_max_ adequately described the efficacy of avibactam when combined with ceftazidime and ceftaroline across the three strains and the researchers recommended to use AUC_0–24_, which could be better estimated in patients as the exposure measure to describe the efficacy of avibactam.^48^

Sy *et al.*^55^ used a semi-mechanistic mathematical model analysis to determine the PK/PD indices of avibactam when combined with ceftazidime against *P. aeruginosa*. They replicated the thigh and lung infection model in neutropenic mice,^40^ presented above, to simulate the relationship between bacterial load and the drug concentrations in the plasma and epithelium of the alveoli following various dose-fractionation studies. The analysis showed that the more frequent dosing regimens of avibactam had a larger killing effect for the same total daily dose, suggesting time-dependent activity, and *fT*_>CT_ was most strongly correlated with a change in bacterial load (R^2 ^= 0.9), with a C_T_ of 1 mg/L.^55^

##### Ceftaroline/avibactam

Louie *et al.*^47^ used an HFIM to evaluate the PD of avibactam in combination with ceftaroline against *K. pneumoniae*, which expresses different β-lactamase enzymes. The dose-ranging studies in this experiment identified that 8 mg/L avibactam administered in combination with ceftaroline (600 mg q8h) consistently succeeded in killing the *K. pneumoniae*. The dose-fractionation study identified *fT*_>CT_ as the PK/PD index linked to the efficacy of avibactam when combined with ceftaroline. However, the study by MacGown *et al*.,^48^ presented above, reported that the efficacy of avibactam when combined with ceftaroline was best described by AUC_0–24_ and *C*_max_.

##### Aztreonam/avibactam

The PK/PD of avibactam in combination with aztreonam was evaluated using an HFIM, and a murine thigh infection model was used to validate the C_T_ of avibactam determined from the HFIM. The efficacy of the combination was assessed against different strains of *E. coli* and *K. pneumoniae*. The HFIM predicted *fT*_>CT_ to be the PK/PD index that best described the efficacy of avibactam when combined with aztreonam, and the C_T_ required for the efficacy of avibactam ranged from 2 to 2.5 mg/L, depending on the type of strain used in both models. The range of *fT*_>CT_ required for net bacterial stasis and 1 log_10_ kill was different for the two models (34%–56.4% and 37.7%–58.2% for stasis and 1 log_10_ kill in the HFIM, and 23%–25% and 35%–40% for stasis and 1 log_10_ kill in the murine thigh infection model).^54^

#### Relebactam

The PK/PD relationship of relebactam (formerly known as MK-7655) in combination with imipenem was explored by using an HFIM and a murine thigh infection model. These studies identified that *f*AUC_0–24_/MIC best described the efficacy of relebactam at the relebactam-potentiated MIC of imipenem.^49,59^ Mavridou *et al.*^49^ reported that the *f*AUC_0–24_/MIC required for stasis effect of relebactam was approximately 5.2, and Wu *et al.*^59^ reported that the *f*AUC_0–24_/MIC (at the MIC of the imipenem/relebactam combination) required for bacterial net stasis, 1 and 2 log_10_ cfu reduction was 8.2, 12 and 18, respectively.

Bhagunde *et al.* used an *in silico* method based on data from the HFIM to determine the PK/PD index of relebactam. The first model relates the PK/PD of imipenem/relebactam to *fT*_>MICi_ at various concentrations of relebactam and >69% *fT*_>MICi_ of the combination is required to suppress microbial growth.^41^ The other model explored *f*AUC_0–24_/MIC (at the MIC of imipenem/relebactam) as a PK/PD index that correlates with the efficacy of relebactam and the magnitude of *f*AUC_0–24_/MIC required for net bacterial stasis, 1 and 2 log kill was 2.5, 4.7 and 7.5, respectively.^42^

#### Vaborbactam

The PK/PD of meropenem and vaborbactam was evaluated by Griffith *et al.*, using an HFIM and mouse thigh infection model against different strains of *E. coli*, *K. pneumoniae* and *E. cloacae*. In both investigations, the PK/PD index that best described the efficacy of vaborbactam was *f*AUC_0–24_/MIC (at the vaborbactam-potentiated meropenem MIC) and the magnitudes of the exposure for net bacterial stasis, 1 and 2 log kill were 12, 18 and 25, respectively, in the HFIM and 9, 38 and 220, respectively, in the mouse thigh infection model.^45^

## Discussion

This systematic review assimilated data on the PK/PD exposure requirements of BLIs when used in combination with BL antibiotics. The major findings of this review are, firstly, the *fT*_>CT_ is the most frequent PK/PD index identified to best describe the efficacy of clavulanic acid, tazobactam and avibactam in terms of enabling sufficient inhibition of β-lactamase enzymes to protect the companion BL antibiotic and thereby achieve either net bacterial stasis or 1 to 2 log kill. For relebactam and vaborbactam, *f*AUC_0–24_/MIC (MIC of the companion BL) is the PK/PD index frequently reported to describe their efficacy. Secondly, the target C_T_ is highly variable from isolate to isolate, depending on the level of β-lactamase enzyme expression and degree of stability of the BL antibiotic against β-lactamase-mediated degradation. The variability and distribution of C_T_ appears to be somehow analogous to the MIC variability and distribution. Thirdly, the proposed magnitude of exposure for the BLI, *fT*_>CT_ and *f*AUC_0–24_/MIC, are not consistent between studies such that existing data appear inadequate to define a generalized target exposure that could be used to guide dosing.

The difference in the PK/PD index identified for BLIs, *fT*_>CT_ for tazobactam,^50–53,56–58^ clavulanic acid^38,46^ and avibactam,^40,43,44,47,54,55^ and *f*AUC_0–24_/MIC for relebactam^42,49,59^ and vaborbactam,^45^ may be related to differences in target binding rate and disassociation kinetics. For instance, tazobactam exhibits relatively slow but irreversible binding,^64^ such that the duration of exposure is important to allow sufficient inhibition; hence the time-dependent PK/PD index, *fT*_>CT_, appears consistent with its binding characteristics. On the other hand, the rapid but slowly reversible target-binding characteristics of vaborbactam^65,66^ mean that the magnitude of exposure over time would facilitate enzyme binding and thus is consistent with the observation of *f*AUC_0–24_/MIC as the PK/PD index related to its efficacy.

The exposure of BLIs required for effective protection is variable among companion BL antibiotics,^25,32,67^ which is in part related to differences in the inherent stability of each BL antibiotic against β-lactamase degradation. Melchers *et al.* determined the C_T_ for tazobactam when combined with two different cephalosporins and found different values: 0.5 mg/L when combined with ceftolozane^50^ and 0.25 mg/L when combined with cefepime.^51^ Furthermore, the magnitude of *fT*_>CT_ required for tazobactam to achieve net bacterial stasis was ≥42%, ≥28% and ≥22% when combined with piperacillin, ceftolozane and cefepime, respectively. This might be related to the relative stability of cefepime against β-lactamase degradation, rapid cell wall penetration and faster bactericidal effect;^68^ as a result, it may need relatively less tazobactam for protection.^33,69^ In another study, Ambrose *et al.*^70^ compared the amount of the BLI CB-618 required to protect the hydrolysis of meropenem and cephalosporins (cefepime, ceftazidime and ceftolozane) against a wide range of β-lactamase enzymes, including OXA-48-like carbapenemase. Exposure–response analysis indicated that a higher CB-618 exposure (*f*AUC_0–24_/MIC) was needed for meropenem relative to that estimated for the cephalosporins as a group from pooled data. This might have been influenced in part by the difference in intrinsic stability of meropenem and cephalosporins against different β-lactamase enzymes such as OXA-48 carbapenemase.^71^

The other factors affecting the magnitude of BLI exposure to protect the companion BL are the β-lactamase enzyme transcription levels and bacterial species. Bacterial strains of the same species, which express a β-lactamase enzyme at different levels (low-, moderate- or high-level expression), require different amounts of BLIs commensurate with the level of expression.^25,52,53,57^ In addition, the exposures of BLIs required to protect the BL against different bacterial species such as *P. aeruginosa*, *K. pneumoniae* and *E. coli* are also different,^46,50,54^ which further implies that the exposure to the BLIs needs to either cover all expected pathogens or be tailored for each isolate.

The high variability in PK/PD exposure requirements of BLIs means that it is challenging to determine a single PK/PD target for multiple bacteria across a wide MIC range. Furthermore, the effects of dynamic BLI concentration on the susceptibility of bacteria to the BL antibiotic has been difficult to integrate into the PK/PD exposure measure. The traditional MIC of the BL/BLI combination is determined at a fixed exposure of BLI that disregards the impact of dynamic inhibitor concentrations on susceptibility.^72–74^ To address this concern, a modified PK/PD index, *fT*_>MICi_ has been proposed for BL/BLI combinations, which reflects the effect of different BLI concentrations on the susceptibility of the bacteria and on how each β-lactamase-producing isolate interacts with the inhibitor.^39,41,74^ However, this target measure remains to be validated against multiple species of bacteria. An alternative approach proposed by other researchers to account for variability is to define C_T_ in *fT*_>CT_ as the product of the individual isolates’ BL/BLI MIC value and a multiplier, which can be easily determined from *in vitro* studies.^56,58^ Nevertheless, the applicability of this approach to various BL/BLI combinations against different bacterial species remains to be validated.

The main purpose of establishing quantifiable PK/PD targets is to use them as a guide for the development and optimization of dosage regimens. However, the PTA for the BLI has rarely been considered in the past, either during drug development or post-marketing optimization, with most studies assessing the PTA for the BL only without considering the PTA for the BLI. Only a few studies have evaluated the appropriateness of the BLI exposure in BL/BLI combinations, mostly for tazobactam (when combined with ceftolozane),^75–80^ avibactam (combined with ceftazidime)^81–83^ and relebactam (when combined with imipenem/cilastatin).^84–87^ The PTA for tazobactam (when combined with ceftolozane, mostly at 1 g/0.5 g q8h) PK/PD target of 20% *fT*_>CT_ at 1 mg/L for stasis,^75–77^ and 35% *fT*_>CT_ at 1 mg/L for 1 log kill,^78^ is greater than 90% in different patient populations.^75–79^ However, tazobactam was unable to attain these PK/PD targets in epithelial lining fluid during dialysis days,^77^ and in CSF, even with the 1.5 g q8h dosage regimen.^80^ This indicates the PK/PD targets of tazobactam might not be achieved in some groups of patients and needs reconsideration. Both ceftazidime/avibactam and imipenem/relebactam had a joint PTA greater than 90% in various patient populations.^81–87^

Similarly, for therapeutic drug monitoring-guided dose optimization of BL/BLI combinations, PK/PD targets for BLIs are usually not considered for dose optimization. Given that all BL/BLI products are available as fixed-dose combinations, adjusting the dose of either the BL or the BLI independently is practically difficult, if not impossible. A potential solution for this clinical challenge may be the use of stand-alone BLIs, simultaneously administered with the BL antibiotic of interest; this will allow greater flexibility to independently tailor the dosage of the BL and the BLI as appropriate for the therapeutic context.^33^

There are some limitations in the studies included in this systematic review. The duration of the experiment for most studies was 24 h, which is insufficient to evaluate the ability of the exposure studied to suppress the regrowth of pre-existing resistant subpopulations, which often emerge during prolonged exposure. In addition, most of the experiments evaluated the magnitudes of the exposure measures at lower bacterial inoculum, which might underestimate the amount of BLI required in severe infections with higher bacterial loads. Testing high inoculums is also advantageous to reveal resistance and study exposures that are likely to suppress regrowth of resistant subpopulations. Additional studies utilizing preclinical infection models (i.e. HFIM) in which the BL/BLI is administered to mimic the anticipated mode(s) of clinical use,^25^ with higher inoculum concentration, are needed to address these limitations.

### Conclusions

*fT*_>CT_ and *f*AUC_0–24_/MIC are useful surrogate indicators of the BLI exposures required for optimal protection of BL antibiotics, ensuring desirable microbiological outcomes. However, the magnitude of exposure required for optimal microbiological outcomes is highly variable depending on the companion BL antibiotic, bacterial species and the level of transcription of genes that express β-lactamase enzyme in the target isolate. As a result, clear inference on what the optimum index is cannot be made. Further PK/PD profiling of BLIs, using dynamic preclinical PK/PD infection models that mimic the clinical dosing/exposure scenario, is therefore warranted to streamline the exposure requirements (targets) for use in optimization of dosing regimens.

## Supplementary Material

dkae058_Supplementary_Data

## Supplementary data

Tables S1 to S5 are available as Supplementary data at *JAC* Online.

## References

[1] Abodakpi H, Wanger A, Tam VH. What the clinical microbiologist should know about pharmacokinetics/pharmacodynamics in the era of emerging multidrug resistance: focusing on β-lactam/β-lactamase inhibitor combinations. Clin Lab Med 2019; 39: 473–85. 10.1016/j.cll.2019.05.006 31383269 PMC6686870

[2] Ruppé É, Woerther P-L, Barbier F. Mechanisms of antimicrobial resistance in Gram-negative bacilli. Ann Intensive Care 2015; 5: 21. 10.1186/s13613-015-0061-0 26261001 PMC4531117

[3] Bush K, Jacoby GA. Updated functional classification of β-lactamases. Antimicrob Agents Chemother 2010; 54: 969–76. 10.1128/AAC.01009-09 19995920 PMC2825993

[4] Tooke CL, Hinchliffe P, Bragginton EC et al β-Lactamases and β-lactamase inhibitors in the 21st century. J Mol Biol 2019; 431: 3472–500. 10.1016/j.jmb.2019.04.002 30959050 PMC6723624

[5] Bush K . Past and present perspectives on β-lactamases. Antimicrob Agents Chemother 2018; 62: e01076-18. 10.1128/AAC.01076-18 30061284 PMC6153792

[6] Lee NLS, Yuen KY, Kumana CR. β-lactam antibiotic and β-lactamase inhibitor combinations. JAMA 2001; 285: 386–8. 10.1001/jama.285.4.386 11242403

[7] Bush K, Bradford PA. β-Lactams and β-lactamase inhibitors: an overview. Cold Spring Harb Perspect Med 2016; 6: a025247. 10.1101/cshperspect.a025247 27329032 PMC4968164

[8] de Sousa Coelho F, Mainardi JL. The multiple benefits of second-generation β-lactamase inhibitors in treatment of multidrug-resistant bacteria. Infectious Diseases Now 2021; 51: 510–7. 10.1016/j.idnow.2020.11.007 33870896

[9] FDA . Ceftazidime-avibactam. Center for Drug Evaluation and Research. Drug Approval Package. 2015. https://www.accessdata.fda.gov/drugsatfda_docs/nda/2015/206494orig1s000toc.cfm.

[10] FDA . FDA Approves New Antibacterial Drug. 2017. https://www.fda.gov/news-events/press-announcements/fda-approves-new-antibacterial-drug.

[11] FDA . FDA Approves Antibiotic to Treat Hospital-Acquired Bacterial Pneumonia and Ventilator-Associated Bacterial Pneumonia. 2020. https://www.fda.gov/news-events/press-announcements/fda-approves-antibiotic-treat-hospital-acquired-bacterial-pneumonia-and-ventilator-associated.

[12] FDA . FDA Approves New Treatment for Hospital-Acquired and Ventilator-Associated Bacterial Pneumonia. 2019. https://www.fda.gov/news-events/press-announcements/fda-approves-new-treatment-hospital-acquired-and-ventilator-associated-bacterial-pneumonia.

[13] Pfizer . Phase 3 Studies of Pfizer’s Novel Antibiotic Combination Offer New Treatment Hope for Patients with Multidrug-Resistant Infections and Limited Treatment Options. 2023. https://www.pfizer.com/news/press-release/press-release-detail/phase-3-studies-pfizers-novel-antibiotic-combination-offer.

[14] Kaye KS, Belley A, Barth P et al Effect of cefepime/enmetazobactam vs piperacillin/tazobactam on clinical cure and microbiological eradication in patients with complicated urinary tract infection or acute pyelonephritis: a randomized clinical trial. JAMA 2022; 328: 1304–14. 10.1001/jama.2022.17034 36194218 PMC9533186

[15] Asempa TE, Kuti JL, Nascimento JC et al Bronchopulmonary disposition of IV cefepime/taniborbactam (2–0.5 g) administered over 2 h in healthy adult subjects. J Antimicrob Chemother 2023; 78: 703–9. 10.1093/jac/dkac447 36617636 PMC9978582

[16] Preston RA, Mamikonyan G, DeGraff S et al Single-center evaluation of the pharmacokinetics of WCK 5222 (cefepime-zidebactam combination) in subjects with renal impairment. Antimicrob Agents Chemother 2019; 63: e01484-18. 10.1128/AAC.01484-18 30397067 PMC6325229

[17] Mallalieu NL, Winter E, Fettner S et al Safety and pharmacokinetic characterization of nacubactam, a novel β-lactamase inhibitor, alone and in combination with meropenem, in healthy volunteers. Antimicrob Agents Chemother 2020; 64: e02229-19. 10.1128/AAC.02229-19 32041717 PMC7179653

[18] Yahav D, Giske CG, Grāmatniece A et al New β-lactam-β-lactamase inhibitor combinations. Clin Microbiol Rev 2020; 34: e00115-20. 10.1128/CMR.00115-20 PMC7667665 33177185

[19] Veeraraghavan B, Bakthavatchalam YD, Kandasamy S et al Clinical efficacy of novel combinations of older beta-lactam and beta-lactamase inhibitors: where are the evidences? Indian J Med Microbiol 2022; 40: 179–81. 10.1016/j.ijmmb.2021.12.005 34972570

[20] Palmer ME, Andrews LJ, Abbey TC et al The importance of pharmacokinetics and pharmacodynamics in antimicrobial drug development and their influence on the success of agents developed to combat resistant gram negative pathogens: a review. Front Pharmacol 2022; 13: 888079. 10.3389/fphar.2022.888079 35959440 PMC9359604

[21] Sime F, Roberts J. PK/PD and the drug delivery regimen for infusion in the critical care setting. In: Berner B, Gordi T, Benson HAE, Roberts MS, eds. Drug Delivery Approaches. Wiley, 2021; 355–74.

[22] Toutain PL, del Castillo JRE, Bousquet-Mélou A. The pharmacokinetic–pharmacodynamic approach to a rational dosage regimen for antibiotics. Res Vet Sci 2002; 73: 105–14. 10.1016/S0034-5288(02)00039-5 12204627

[23] Lister PD . The role of pharmacodynamic research in the assessment and development of new antibacterial drugs. Biochem Pharmacol 2006; 71: 1057–65. 10.1016/j.bcp.2005.10.038 16316633

[24] Sime FB, Roberts JA. Antibiotic pharmacodynamics. In: Udy AA, Roberts JA, Lipman J, eds. Antibiotic Pharmacokinetic/Pharmacodynamic Considerations in the Critically Ill. Springer Nature, 2018; 17–29.

[25] EMA . Use of Pharmacokinetics and Pharmacodynamics in the Development of Antimicrobial Medicinal Products – Scientific Guideline. 2016. https://www.ema.europa.eu/en/use-pharmacokinetics-and-pharmacodynamics-development-antibacterial-medicinal-products-scientific-guideline.

[26] Rodríguez-Gascón A, Solinís M, Isla A. The role of PK/PD analysis in the development and evaluation of antimicrobials. Pharmaceutics 2021; 13: 833. 10.3390/pharmaceutics13060833 34205113 PMC8230268

[27] Nielsen EI, Cars O, Friberg LE. Pharmacokinetic/pharmacodynamic (PK/PD) indices of antibiotics predicted by a semimechanistic PKPD model: a step toward model-based dose optimization. Antimicrob Agents Chemother 2011; 55: 4619–30. 10.1128/AAC.00182-11 21807983 PMC3186970

[28] Sou T, Hansen J, Liepinsh E et al Model-informed drug development for antimicrobials: translational PK and PK/PD modeling to predict an efficacious human dose for apramycin. Clin Pharmacol Ther 2021; 109: 1063–73. 10.1002/cpt.2104 33150591 PMC8048880

[29] Tängdén T, Lundberg CV, Friberg LE et al How preclinical infection models help define antibiotic doses in the clinic. Int J Antimicrob Agents 2020; 56: 106008. 10.1016/j.ijantimicag.2020.106008 32389722

[30] VanScoy BD, Trang M, McCauley J et al Pharmacokinetics-pharmacodynamics of a novel β-lactamase inhibitor, CB-618, in combination with meropenem in an in vitro infection model. Antimicrob Agents Chemother 2016; 60: 3891–6. 10.1128/AAC.02943-15 27001820 PMC4914694

[31] Onufrak NJ, Forrest A, Gonzalez D. Pharmacokinetic and pharmacodynamic principles of anti-infective dosing. Clin Ther 2016; 38: 1930–47. 10.1016/j.clinthera.2016.06.015 27449411 PMC5039113

[32] Berry AV, Kuti JL. Pharmacodynamic thresholds for beta-lactam antibiotics: a story of mouse versus man. Front Pharmacol 2022; 13: 833189. 10.3389/fphar.2022.833189 35370708 PMC8971958

[33] Crass RL, Pai MP. Pharmacokinetics and pharmacodynamics of β-lactamase inhibitors. Pharmacotherapy 2019; 39: 182–95. 10.1002/phar.2210 30589457

[34] Page MJ, McKenzie JE, Bossuyt PM et al The PRISMA 2020 statement: an updated guideline for reporting systematic reviews. BMJ 2021; 372: n71. 10.1136/bmj.n71 33782057 PMC8005924

[35] Nendza M, Aldenberg T, Benfenati E et al Chapter 4. Data quality assessment for in silico methods: a survey of approaches and needs. In: Cronin MTD, Madden JC, eds. In Silico Toxicology. Royal Society of Chemistry, 2010.

[36] Schneider K, Schwarz M, Burkholder I et al “ToxRTool”, a new tool to assess the reliability of toxicological data. Toxicol Lett 2009; 189: 138–44. 10.1016/j.toxlet.2009.05.013 19477248

[37] Sumi CD, Heffernan AJ, Lipman J et al What antibiotic exposures are required to suppress the emergence of resistance for Gram-negative bacteria? A systematic review. Clin Pharmacokinet 2019; 58: 1407–43. 10.1007/s40262-019-00791-z 31325141

[38] Abdelraouf K, Stainton SM, Nicolau DP. *In vivo* pharmacodynamic profile of ceftibuten-clavulanate combination against extended-spectrum-β-lactamase-producing Enterobacteriaceae in the murine thigh infection model. Antimicrob Agents Chemother 2019; 63: e00145-19. 10.1128/AAC.00145-19 31061165 PMC6591625

[39] Abodakpi H, Chang KT, Gao S et al Optimal piperacillin-tazobactam dosing strategies against extended-spectrum-β-lactamase-producing Enterobacteriaceae. Antimicrob Agents Chemother 2019; 63: e01906-18. 10.1128/AAC.01906-18 30530606 PMC6355564

[40] Berkhout J, Melchers MJ, van Mil AC et al Pharmacodynamics of ceftazidime and avibactam in neutropenic mice with thigh or lung infection. Antimicrob Agents Chemother 2016; 60: 368–75. 10.1128/AAC.01269-15 26525790 PMC4704241

[41] Bhagunde P, Chang KT, Hirsch EB et al Novel modeling framework to guide design of optimal dosing strategies for β-lactamase inhibitors. Antimicrob Agents Chemother 2012; 56: 2237–40. 10.1128/AAC.06113-11 22330927 PMC3346582

[42] Bhagunde P, Zhang Z, Racine F et al A translational pharmacokinetic/pharmacodynamic model to characterize bacterial kill in the presence of imipenem-relebactam. Int J Infect Dis 2019; 89: 55–61. 10.1016/j.ijid.2019.08.026 31479762

[43] Coleman K, Levasseur P, Girard AM et al Activities of ceftazidime and avibactam against β-lactamase-producing Enterobacteriaceae in a hollow-fiber pharmacodynamic model. Antimicrob Agents Chemother 2014; 58: 3366–72. 10.1128/AAC.00080-14 24687507 PMC4068505

[44] Drusano GL, Shields RK, Mtchedlidze N et al Pharmacodynamics of ceftazidime plus avibactam against KPC-2-bearing isolates of *Klebsiella pneumoniae* in a hollow fiber infection model. Antimicrob Agents Chemother 2019; 63: e00462-19. 10.1128/AAC.00462-19 31160285 PMC6658759

[45] Griffith DC, Sabet M, Tarazi Z et al Pharmacokinetics/pharmacodynamics of vaborbactam, a novel beta-lactamase inhibitor, in combination with meropenem. Antimicrob Agents Chemother 2019; 63: e01659-18. 10.1128/AAC.01659-18 30397063 PMC6325214

[46] Grupper M, Stainton SM, Nicolau DP et al *In vitro* pharmacodynamics of a novel ceftibuten-clavulanate combination antibiotic against Enterobacteriaceae. Antimicrob Agents Chemother 2019; 63: e00144-19. 10.1128/AAC.00144-19 31061148 PMC6591603

[47] Louie A, Castanheira M, Liu W et al Pharmacodynamics of β-lactamase inhibition by NXL104 in combination with ceftaroline: examining organisms with multiple types of β-lactamases. Antimicrob Agents Chemother 2012; 56: 258–70. 10.1128/AAC.05005-11 22024819 PMC3256033

[48] MacGowan A, Tomaselli S, Noel A et al The pharmacodynamics of avibactam in combination with ceftaroline or ceftazidime against β-lactamase-producing Enterobacteriaceae studied in an *in vitro* model of infection. J Antimicrob Chemother 2017; 72: 762–9. 10.1093/jac/dkw480 28039276

[49] Mavridou E, Melchers RJ, van Mil AC et al Pharmacodynamics of imipenem in combination with β-lactamase inhibitor MK7655 in a murine thigh model. Antimicrob Agents Chemother 2015; 59: 790–5. 10.1128/AAC.03706-14 25403667 PMC4335905

[50] Melchers MJ, Mavridou E, van Mil AC et al Pharmacodynamics of ceftolozane combined with tazobactam against Enterobacteriaceae in a neutropenic mouse thigh model. Antimicrob Agents Chemother 2016; 60: 7272–9. 10.1128/AAC.01580-16 27671063 PMC5119007

[51] Melchers MJ, van Mil AC, Lagarde C et al Pharmacodynamics of cefepime combined with tazobactam against clinically relevant Enterobacteriaceae in a neutropenic mouse thigh model. Antimicrob Agents Chemother 2017; 61: e00267-17. 10.1128/AAC.00267-17 28630197 PMC5571283

[52] Nicasio AM, VanScoy BD, Mendes RE et al Pharmacokinetics-pharmacodynamics of tazobactam in combination with piperacillin in an *in vitro* infection model. Antimicrob Agents Chemother 2016; 60: 2075–80. 10.1128/AAC.02747-15 26787689 PMC4808219

[53] Rodriguez CA, Agudelo M, Zuluaga AF et al In vivo pharmacodynamics of piperacillin/tazobactam: implications for antimicrobial efficacy and resistance suppression with innovator and generic products. Int J Antimicrob Agents 2017; 49: 189–97. 10.1016/j.ijantimicag.2016.10.011 27988068

[54] Singh R, Kim A, Tanudra MA et al Pharmacokinetics/pharmacodynamics of a β-lactam and β-lactamase inhibitor combination: a novel approach for aztreonam/avibactam. J Antimicrob Chemother 2015; 70: 2618–26. 10.1093/jac/dkv132 26024868

[55] Sy SKB, Zhuang L, Xia H et al A model-based analysis of pharmacokinetic-pharmacodynamic (PK/PD) indices of avibactam against *Pseudomonas aeruginosa*. Clin Microbiol Infect 2019; 25: 904.e9–e16. 10.1016/j.cmi.2018.10.014 30394361

[56] VanScoy B, Mendes RE, McCauley J et al Pharmacological basis of β-lactamase inhibitor therapeutics: tazobactam in combination with ceftolozane. Antimicrob Agents Chemother 2013; 57: 5924–30. 10.1128/AAC.00656-13 24041895 PMC3837916

[57] VanScoy B, Mendes RE, Nicasio AM et al Pharmacokinetics-pharmacodynamics of tazobactam in combination with ceftolozane in an *in vitro* infection model. Antimicrob Agents Chemother 2013; 57: 2809–14. 10.1128/AAC.02513-12 23629705 PMC3716129

[58] VanScoy BD, Tenero D, Turner S et al Pharmacokinetics-pharmacodynamics of tazobactam in combination with cefepime in an *in vitro* infection model. Antimicrob Agents Chemother 2017; 61: e01052-17. 10.1128/AAC.01052-17 28947475 PMC5700300

[59] Wu J, Racine F, Wismer MK et al Exploring the pharmacokinetic/pharmacodynamic relationship of relebactam (MK-7655) in combination with imipenem in a hollow-fiber infection model. Antimicrob Agents Chemother 2018; 62: e02323-17. 10.1128/AAC.02323-17 29507068 PMC5923117

[60] Yokoyama Y, Matsumoto K, Ikawa K et al Pharmacokinetic/pharmacodynamic evaluation of sulbactam against *Acinetobacter baumannii* in *in vitro* and murine thigh and lung infection models. Int J Antimicrob Agents 2014; 43: 547–52. 10.1016/j.ijantimicag.2014.02.012 24796218

[61] Nichols WW, Newell P, Critchley IA et al Avibactam pharmacokinetic/pharmacodynamic targets. Antimicrob Agents Chemother 2018; 62: e02446-17. 10.1128/AAC.02446-17 29610208 PMC5971577

[62] Alexov M, Lister PD, Sanders CC. Efficacy of ampicillin-sulbactam is not dependent upon maintenance of a critical ratio between components: sulbactam pharmacokinetics in pharmacodynamic interactions. Antimicrob Agents Chemother 1996; 40: 2468–77. 10.1128/AAC.40.11.2468 8913448 PMC163559

[63] Lister PD, Prevan AM, Sanders CC. Importance of β-lactamase inhibitor pharmacokinetics in the pharmacodynamics of inhibitor-drug combinations: studies with piperacillin-tazobactam and piperacillin-sulbactam. Antimicrob Agents Chemother 1997; 41: 721–7. 10.1128/AAC.41.4.721 9087477 PMC163782

[64] Carcione D, Siracusa C, Sulejmani A et al Old and new beta-lactamase inhibitors: molecular structure, mechanism of action, and clinical use. Antibiotics (Basel) 2021; 10: 995. 10.3390/antibiotics10080995 34439045 PMC8388860

[65] Lomovskaya O, Sun D, Rubio-Aparicio D et al Vaborbactam: spectrum of beta-lactamase inhibition and impact of resistance mechanisms on activity in Enterobacteriaceae. Antimicrob Agents Chemother 2017; 61: e01443-17. 10.1128/AAC.01443-17 28848018 PMC5655098

[66] Novelli A, Del Giacomo P, Rossolini GM et al Meropenem/vaborbactam: a next generation β-lactam β-lactamase inhibitor combination. Expert Rev Anti Infect Ther 2020; 18: 643–55. 10.1080/14787210.2020.1756775 32297801

[67] Monogue ML, Nicolau DP. Pharmacokinetics-pharmacodynamics of β-lactamase inhibitors: are we missing the target? Expert Rev Anti Infect Ther 2019; 17: 571–82. 10.1080/14787210.2019.1647781 31340665

[68] Livermore DM, Mushtaq S, Warner M et al Potential of high-dose cefepime/tazobactam against multiresistant Gram-negative pathogens. J Antimicrob Chemother 2017; 73: 126–33. 10.1093/jac/dkx360 29059308

[69] Livermore D . Determinants of the activity of β-lactamase inhibitor combinations. J Antimicrob Chemother 1993; 31: 9–21. 10.1093/jac/31.suppl_A.9 8449836

[70] Ambrose PG, VanScoy BD, Trang M et al Pharmacokinetics-pharmacodynamics of CB-618 in combination with cefepime, ceftazidime, ceftolozane, or meropenem: the pharmacological basis for a stand-alone β-lactamase inhibitor. Antimicrob Agents Chemother 2017; 61: e00630-17. 10.1128/AAC.00630-17 28947474 PMC5700306

[71] Poirel L, Potron A, Nordmann P. OXA-48-like carbapenemases: the phantom menace. J Antimicrob Chemother 2012; 67: 1597–606. 10.1093/jac/dks121 22499996

[72] Dingle TC, Pitout J. The ins and outs of susceptibility testing for new β-lactam/β-lactamase inhibitor combinations for Gram-negative organisms. J Clin Microbiol 2022; 60: e00807-21. 10.1128/jcm.00807-21 35387484 PMC9297814

[73] Brown D . Susceptibility Testing β-Lactamase Inhibitor Combinations. 2016. https://bsac.org.uk/wp-content/uploads/2016/01/5-DB-BSAC-user-group-2016f.pdf.

[74] Tam VH, Abodakpi H, Wang W et al Optimizing pharmacokinetics/pharmacodynamics of β-lactam/β-lactamase inhibitor combinations against high inocula of ESBL-producing bacteria. J Antimicrob Chemother 2021; 76: 179–83. 10.1093/jac/dkaa412 33035321 PMC7729384

[75] Sime FB, Lassig-Smith M, Starr T et al Population pharmacokinetics of unbound ceftolozane and tazobactam in critically ill patients without renal dysfunction. Antimicrob Agents Chemother 2019; 63: e01265-19. 10.1128/AAC.01265-19 31358583 PMC6761554

[76] Xiao AJ, Caro L, Popejoy MW et al PK/PD target attainment with ceftolozane/tazobactam using Monte Carlo simulation in patients with various degrees of renal function, including augmented renal clearance and end-stage renal disease. Infect Dis Ther 2017; 6: 137–48. 10.1007/s40121-016-0143-9 28013453 PMC5336418

[77] Feng HP, Patel YT, Zhang Z et al Probability of target attainment analyses to inform ceftolozane/tazobactam dosing regimens for patients with hospital-acquired or ventilator-associated bacterial pneumonia and end-stage renal disease receiving intermittent hemodialysis. J Clin Pharmacol 2023; 63: 166–71. 10.1002/jcph.2149 36046982 PMC10092127

[78] Gao W, Patel YT, Zhang Z et al Ceftolozane/tazobactam probability of target attainment in patients with hospital-acquired or ventilator-associated bacterial pneumonia. J Clin Pharmacol 2023; 63: 352–7. 10.1002/jcph.2165 36201105

[79] Shorr AF, Bruno CJ, Zhang Z et al Ceftolozane/tazobactam probability of target attainment and outcomes in participants with augmented renal clearance from the randomized phase 3 ASPECT-NP trial. Crit Care 2021; 25: 354. 10.1186/s13054-021-03773-5 34600585 PMC8487337

[80] Sime FB, Lassig-Smith M, Starr T et al Cerebrospinal fluid penetration of ceftolozane-tazobactam in critically ill patients with an indwelling external ventricular drain. Antimicrob Agents Chemother 2020; 65: e01698-20. 10.1128/AAC.01698-20 33077655 PMC7927828

[81] Bensman TJ, Wang J, Jayne J et al Pharmacokinetic-pharmacodynamic target attainment analyses to determine optimal dosing of ceftazidime-avibactam for the treatment of acute pulmonary exacerbations in patients with cystic fibrosis. Antimicrob Agents Chemother 2017; 61: e00988-17. 10.1128/AAC.00988-17 28784670 PMC5610479

[82] Franzese RC, McFadyen L, Watson KJ et al Population pharmacokinetic modeling and probability of pharmacodynamic target attainment for ceftazidime-avibactam in pediatric patients aged 3 months and older. Clin Pharmacol Ther 2022; 111: 635–45. 10.1002/cpt.2460 34687548 PMC9298731

[83] Li J, Lovern M, Green ML et al Ceftazidime-avibactam population pharmacokinetic modeling and pharmacodynamic target attainment across adult indications and patient subgroups. Clin Transl Sci 2019; 12: 151–63. 10.1111/cts.12585 30221827 PMC6440567

[84] Patel M, Bellanti F, Daryani NM et al Population pharmacokinetic/pharmacodynamic assessment of imipenem/cilastatin/relebactam in patients with hospital-acquired/ventilator-associated bacterial pneumonia. Clin Transl Sci 2022; 15: 396–408. 10.1111/cts.13158 34704389 PMC8841461

[85] Bhagunde P, Patel P, Lala M et al Population pharmacokinetic analysis for imipenem-relebactam in healthy volunteers and patients with bacterial infections. CPT Pharmacometrics Syst Pharmacol 2019; 8: 748–58. 10.1002/psp4.12462 31508899 PMC6813166

[86] Fratoni AJ, Mah JW, Nicolau DP et al Imipenem/cilastatin/relebactam pharmacokinetics in critically ill patients with augmented renal clearance. J Antimicrob Chemother 2022; 77: 2992–9. 10.1093/jac/dkac261 35906810

[87] Roberts JA, Nicolau DP, Martin-Loeches I et al Imipenem/cilastatin/relebactam efficacy, safety and probability of target attainment in adults with hospital-acquired or ventilator-associated bacterial pneumonia among patients with baseline renal impairment, normal renal function, and augmented renal clearance. JAC Antimicrob Resist 2023; 5: dlad011. 10.1093/jacamr/dlad011 36880088 PMC9985325

