# Supplementary material for: What are the optimal pharmacokinetic/pharmacodynamic targets for β-lactamase inhibitors? A systematic review
Source: J Antimicrob Chemother. 2024 Mar 9;79(5):946–58. doi: 10.1093/jac/dkae058 (PMC11062945; doi:10.1093/jac/dkae058)
Supplement: dkae058_Supplementary_Data [file dkae058_supplementary_data.docx]

**Supplementary material of “What are the optimal pharmacokinetic/ pharmacodynamic targets for β-lactamase inhibitors? A systematic review”**

Getnet M Assefa, Jason A Roberts, Solomon A Mohammed, Fekade B Sime

Table S1 Search strategies used to retrieve articles from different databases

| ***Databases*** | ***Search Terms*** |
| --- | --- |
| Scopus | ((TITLE-ABS-KEY("Beta-lactamase inhibitor*") OR TITLE-ABS-KEY("Clavulanic acid") OR TITLE-ABS-KEY(Tazobactam) OR TITLE-ABS-KEY(Sulbactam) OR TITLE-ABS-KEY(Avibactam) OR TITLE-ABS-KEY(Vaborbactam) OR TITLE-ABS-KEY(Relebactam))) AND ((TITLE-ABS-KEY("Target exposure") OR TITLE-ABS-KEY("Target concentration") OR TITLE-ABS-KEY("Threshold concentration") OR TITLE-ABS-KEY("PK/PD index") OR TITLE-ABS-KEY("PK/PD target") OR TITLE-ABS-KEY("PK/PD ratio") OR TITLE-ABS-KEY(pharmacodynamic*) OR TITLE-ABS-KEY(IC50) OR TITLE-ABS-KEY("Effective concentration") OR TITLE-ABS-KEY("Pharmacokinetic*/Pharmacodynamic*") OR TITLE-ABS-KEY("Critical concentration") OR TITLE-ABS-KEY("PD inde*"))) AND ( LIMIT-TO ( DOCTYPE,"ar" ) OR LIMIT-TO ( DOCTYPE,"cp" ) ) AND ( LIMIT-TO ( LANGUAGE,"English" ) ) |
| Web of Science | (((((((TS=("beta lactamase inhibitor*")) OR TS=(“Clavulanic acid”)) OR TS=(tazobactam)) OR TS=(sulbactam)) OR TS=(avibactam)) OR TS=(vaborbactam)) OR TS=(relebactam)) AND ((((((((((((TS=(“Target exposure”)) OR TS=(“Target concentration”)) OR TS=(“Threshold concentration”)) OR TS=(“PK/PD index”)) OR TS=(“PK/PD target”)) OR TS=(“PK/PD ratio”)) OR TS=(pharmacodynamic*)) OR TS=(IC50)) OR TS=(“Effective concentration”)) OR TS=(“Pharmacokinetic*/Pharmacodynamic*”)) OR TS=(“Critical concentration”)) OR TS=(“PD inde*”)); Document types; Article or proceeding paper or early access, language: English |
| PubMed | (((((((Beta lactamase inhibitor[MeSH]) OR ("Clavulanic acid"[Title/Abstract])) OR (Tazobactam[Title/Abstract])) OR (Sulbactam[Title/Abstract])) OR (Avibactam[Title/Abstract])) OR (Vaborbactam[Title/Abstract])) OR (Relebactam[Title/Abstract])) AND (((((((((((("Target exposure"[Title/Abstract]) OR ("Target concentration"[Title/Abstract])) OR ("Threshold concentration"[Title/Abstract])) OR ("PK/PD index"[Title/Abstract])) OR ("PK/PD target"[Title/Abstract])) OR (PK/PD ratio[Title/Abstract])) OR (Pharmacodynamic*[Title/Abstract])) OR (IC50[Title/Abstract])) OR ("Effective concentration"[Title/Abstract])) OR ("Pharmacokinetic*/Pharmacodynamic*"[Title/Abstract])) OR ("Critical concentration"[Title/Abstract])) OR ("PD inde*"[Title/Abstract])) AND (english[Filter]) |
| Embase | ('beta lactamase inhibitor*':ti,ab,kw OR 'clavulanic acid':ti,ab,kw OR tazobactam:ti,ab,kw OR sulbactam:ti,ab,kw OR avibactam:ti,ab,kw OR vaborbactam:ti,ab,kw OR relebactam:ti,ab,kw) AND ('target exposure':ti,ab,kw OR 'target concentration':ti,ab,kw OR 'threshold concentration':ti,ab,kw OR 'pk/pd index':ti,ab,kw OR 'pharmacokinetic*/pharmacodynamic* index':ti,ab,kw OR 'pk/pd target':ti,ab,kw OR 'pharmacokinetic*/pharmacodynamic* target':ti,ab,kw OR 'pk/pd ratio':ti,ab,kw OR 'pharmacokinetic*/pharmacodynamic* ratio':ti,ab,kw OR pharmacodynamic*:ti,ab,kw OR ic50:ti,ab,kw OR 'effective concentration':ti,ab,kw OR 'pharmacokinetic*/pharmacodynamic*':ti,ab,kw OR 'critical concentration':ti,ab,kw OR 'pd inde*':ti,ab,kw) AND ('article'/it OR 'article in press'/it OR 'conference paper'/it) AND [english]/lim |
| Cochrane Library | (("Beta lactamase inhibitor*"):ti,ab,kw OR ("Clavulanic acid"):ti,ab,kw OR (Tazobactam) :ti,ab,kw OR (Sulbactam):ti,ab,kw OR (Avibactam):ti,ab,kw OR (Vaborbactam):ti,ab,kw OR (Relebactam):ti,ab,kw) AND ((“Target exposure”):ti,ab,kw OR (“Target concentration”)ti,ab,kw OR (“PK PD index”):ti,ab,kw OR (“PK PD ratio”):ti,ab,kw OR (“PK PD target”):ti,ab,kw OR (pharmacodynamic*):ti,ab,kw OR (IC50):ti,ab,kw OR (“Effective concentration”):ti,ab,kw OR (“Pharmacokinetic Pharmacodynamic”):ti,ab,kw OR (“Critical concentration”):ti,ab,kw OR (“PD index”):ti,ab,kw) |

Table S2 Quality assessment tool for the in vitro and in vivo Studies

| **Category of items** | **Sub items** | **Score**  **(No =0, Yes=1)** |
| --- | --- | --- |
| Aim of the study | 1. Was the research question of the study clearly stated? |  |
| Materials and Methods | 1. Were the names and sources of test drugs clearly stated? |  |
|  | 1. Was the dosing regimen of test drugs clearly described? |  |
|  | 1. Were the species of the bacteria used in the study reported? |  |
|  | 1. Were the types of strains of the bacteria reported? |  |
|  | 1. Was the baseline bacterial concentration used clearly stated? |  |
|  | 1. Were the types of beta-lactamase enzymes expressed clearly stated? |  |
|  | 1. Was the type of *in vitro* and/or *in vivo* model used clearly described? |  |
|  | 1. Was the dose fractionation study clearly described? |  |
|  | 1. Was growth control included? |  |
|  | 1. Was the study observation's duration and/or time point clearly described? |  |
|  | 1. Was the test drug concentration measurement method validated? |  |
|  | 1. Was the outcome analyzed with appropriate statistical methods? |  |
| Outcomes | 1. Was the exposure measure associated with the efficacy of β-lactamase inhibitor clearly stated? |  |
|  | 1. Were the magnitudes of the exposure measures necessary for the efficacy of β-lactamase inhibitor clearly described? |  |

Table S3 Quality Assessment tool for the in-silico Studies

| **Category of items** | **Sub items** | **Score**  **(No =0, Yes=1)** |
| --- | --- | --- |
| Aim of the study | 1. Was the research question of the study clearly stated? |  |
| Materials and Methods | 1. Were the names and sources of test drugs clearly stated? |  |
|  | 1. Was the dosing regimen of test drugs clearly simulated in the model? |  |
|  | 1. Were the species of the bacteria used in the study reported? |  |
|  | 1. Were the types of β-lactamase enzymes expressed clearly stated? |  |
|  | 1. Was the pharmacokinetic-pharmacodynamic model used validated? |  |
|  | 1. Was the *in-silico* dose fractionation simulation clearly described? |  |
|  | 1. Was the baseline population (type of bacteria) used for method development similar to the bacteria for which PK/PD target was estimated? |  |
|  | 1. Was the study observation's duration and/or time point clearly described? |  |
|  | 1. Was the outcome analyzed with appropriate statistical methods? 2. Were the *in-silico* predictions prospectively validated? |  |
| Outcomes | 1. Was the appropriate methodology followed to determine the exposure measure associated with the efficacy of the β-lactamase inhibitor? |  |
|  | 1. Were the magnitudes of the exposure measures necessary for the efficacy of β-lactamase inhibitor clearly described? |  |

Table S4 Quality assessment results for in vitro and in vivo studies included in the systematic review

| **Name & Year** | Item 1 | Item 2 | Item 3. | Item 4. | Item 5. | Item 6. | Item 7. | Item 8. | Item 9. | Item 10. | Item 11. | Item 12. | Item 13. | Item 14. | Item 15. | yes score |
| --- | --- | --- | --- | --- | --- | --- | --- | --- | --- | --- | --- | --- | --- | --- | --- | --- |
| Abdelraouf *et al.*, 2019 | yes | yes | yes | yes | yes | yes | yes | yes | yes | yes | yes | no | yes | yes | yes | 14 |
| Abodakpi *et al.*, 2019a | yes | yes | yes | yes | yes | yes | yes | yes | yes | yes | yes | yes | yes | yes | no | 14 |
| Berkhout *et al.*, 2016 | yes | yes | yes | yes | yes | yes | yes | yes | yes | yes | yes | yes | yes | yes | yes | 15 |
| Coleman *et al.*, 2014 | yes | yes | yes | yes | yes | yes | yes | yes | no | yes | yes | yes | yes | yes | no | 13 |
| Drusano *et al.*, 2019 | yes | yes | yes | yes | yes | yes | yes | yes | no | yes | yes | yes | yes | yes | no | 13 |
| Griffith *et al.*, 2019 | yes | yes | yes | yes | yes | yes | yes | yes | yes | yes | yes | yes | yes | yes | yes | 15 |
| Grupper *et al.*, 2019 | yes | yes | yes | yes | yes | yes | yes | yes | yes | yes | yes | yes | yes | yes | no | 14 |
| Louie *et al.*, 2012 | yes | yes | yes | yes | yes | yes | yes | yes | no | yes | yes | yes | yes | yes | no | 13 |
| MacGowan *et al.*, 2017 | yes | yes | yes | yes | yes | no | yes | yes | no | yes | yes | yes | yes | yes | no | 12 |
| Mavridou *et al.*, 2015 | yes | yes | yes | yes | yes | yes | yes | yes | no | yes | yes | yes | yes | yes | no | 13 |
| Melchers *et al.*, 2016 | yes | yes | yes | yes | yes | yes | yes | yes | yes | yes | yes | yes | yes | yes | yes | 15 |
| Melchers *et al.*, 2017 | yes | yes | yes | yes | yes | yes | yes | yes | yes | yes | yes | yes | yes | yes | yes | 15 |
| Nicasio *et al.*, 2016 | yes | yes | yes | yes | yes | yes | yes | yes | yes | yes | yes | yes | yes | yes | yes | 15 |
| Rodriguez *et al.*, 2017 | yes | yes | yes | yes | yes | yes | yes | yes | no | yes | yes | yes | yes | yes | yes | 14 |
| Singh *et al.*, 2015 | yes | yes | yes | yes | yes | yes | yes | yes | yes | yes | yes | yes | yes | yes | yes | 15 |
| Vanscoy *et al.*, 2013a | yes | yes | yes | yes | yes | yes | yes | yes | yes | yes | yes | yes | yes | yes | yes | 15 |
| VanScoy *et al.*, 2013b | yes | yes | yes | yes | yes | yes | yes | yes | yes | yes | yes | yes | yes | yes | yes | 15 |
| VanScoy *et al.*, 2017 | yes | yes | yes | yes | yes | yes | yes | yes | yes | yes | yes | yes | yes | yes | yes | 15 |
| Wu *et al.*, 2018 | yes | yes | yes | yes | yes | yes | yes | yes | yes | yes | yes | yes | yes | yes | yes | 15 |
| Yokoyama *et al.*, 2014 | yes | yes | yes | yes | yes | yes | no | yes | yes | yes | yes | yes | yes | yes | yes | 14 |

Item 1; Was the research question of the study clearly stated?

Item 2; Were the name and sources of test drugs clearly stated?

Item 3; Were the dosing regimen of test drugs clearly described?

Item 4; Were the species of the bacteria used in the study reported?

Item 5; Were the types of strains of the bacteria reported?

Item 6; Was the baseline bacterial concentration used clearly stated?

Item 7; Were the types of β-lactamase enzymes expressed clearly stated?

Item 8; Was the type of *in vitro* and/or *in vivo* model used clearly described?

Item 9; Was the dose fractionation/ranging study clearly described?

Item 10; Was growth control included?

Item 11; Was the study observation's duration and/or time point clearly described?

Item 12; Was the test drug concentration measurement method validated?

Item 13; Was the outcome analysed with appropriate statistical methods?

Item 14; Was the exposure measure associated with the efficacy of β-lactamase inhibitor clearly stated?

Item 15; Were the magnitudes of the exposure measures necessary for the efficacy of β-lactamase inhibitor clearly described?

Supplementary Table 5 Quality assessment results for in silico studies included in the systematic review

| **Name & Year** | Item 1 | Item 2 | Item 3. | Item 4. | Item 5. | Item 6. | Item 7. | Item 8. | Item 9. | Item 10. | Item 11. | Item 12. | Item 13. | yes score |
| --- | --- | --- | --- | --- | --- | --- | --- | --- | --- | --- | --- | --- | --- | --- |
| Bhagunde et al., 2012 | Yes | Yes | Yes | Yes | Yes | Yes | Yes | Yes | Yes | Yes | Yes | Yes | No | 12 |
| Bhagunde et al., 2019 | Yes | Yes | Yes | Yes | No | Yes | Yes | Yes | Yes | Yes | No | Yes | Yes | 11 |
| Sy et al., 2019 | Yes | Yes | Yes | Yes | Yes | Yes | Yes | Yes | Yes | Yes | No | Yes | Yes | 12 |

Item 1; Was the research question of the study clearly stated?

Item 2; Were the names and sources of test drugs clearly stated?

Item 3; Was the dosing regimen of test drugs clearly simulated in the model?

Item 4; Were the species of the bacteria used in the study reported?

Item 5; Were the types of β-lactamase enzymes expressed clearly stated?

Item 6; Was the pharmacokinetic-pharmacodynamic model used validated?

Item 7; Was the in-silico dose fractionation simulation clearly described?

Item 8; Was the baseline population (type of bacteria) used for method development similar to the bacteria for which PK/PD target was estimated?

Item 9; Was the study observation's duration and/or time point clearly described?

Item 10; Was the outcome analysed with appropriate statistical methods?

Item 11; Were the in-silico predictions prospectively validated?

Item 12; Was the appropriate methodology followed to determine the exposure measure associated with the efficacy of the β-lactamase inhibitor?

Item 13; Were the magnitudes of the exposure measures necessary for the efficacy of β-lactamase inhibitor clearly described?
